# Impact of an educational intervention regarding tobacco counseling on dentists and dental students

**DOI:** 10.1590/1807-3107bor-2024.vol38.0102

**Published:** 2024-11-08

**Authors:** Tiago Luís Herpich, Eduarda Martins Mendes, Michelle Roxo-Gonçalves, Natan Katz, Janete Dias Almeida, Manoela Domingues Martins, Juliana Romanini, Vinicius Coelho Carrard

**Affiliations:** (a) Universidade Federal do Rio Grande do Sul – UFRGS, School of Dentistry, Department of Oral Pathology, Porto Alegre, RS, Brazil.; (b) Universidade Federal do Rio Grande do Sul – UFRGS, TelessaúdeRS-UFRGS, Porto Alegre, RS, Brazil.; (c) Universidade Estadual Paulista – Unesp, Institute of Science and Technology, Department of Biosciences and Diagnosis, São José dos Campos, SP, Brazil.; (d) Porto Alegre City Hall, Centro de Especialidades Odontológicas, Estomatologia, Porto Alegre, RS, Brazil

**Keywords:** Education, Dental, Continuing, Oral Health, Tobacco Use Cessation

## Abstract

The present study aimed to assess the knowledge, attitudes, and perceptions of dental professionals and students regarding tobacco cessation counseling (TCC) after their participation in a continuing education activity (CEA) entitled "Smoking cessation: How does the dentist participate in this decision?" at the Oral Cancer Seminar: Projeto Maio Vermelho 2021. This study utilized a pre-/post-intervention design, including a pre-intervention questionnaire with 20 close-ended questions, an educational intervention, and a post-intervention questionnaire with nine close-ended questions. Descriptive and statistical analyses were performed using SPSS 25 and GraphPad Prism 8 software. The significance level was set at *p* = 0.05. A total of 94 participants answered the pre-intervention questionnaire and 52 answered both the pre- and post-intervention questionnaires. Most participants reported regularly asking about smoking status (96.8%), providing advice on tobacco risks (96.8%), and offering some counseling to help patients stop smoking (84.0%). Although participants habitually ask about cigarette use, other forms of tobacco consumption are frequently overlooked. Most participants reported never having attended TCC training during their undergraduate studies (67.0%) or after graduation (71.2%). However, 96.2% showed interest in attending TCC training. The perception that motivational counseling by dentists can encourage patients to stop smoking rose from 87.5 to 98.2% (p<0.05) after the educational intervention. In addition, participants’ self-confidence in conducting TCC increased from 8.9% to 23.3% (p<0.01). The brief CEA on TCC showed favorable outcomes, enhancing the perception of dentists and undergraduate dental students regarding the effectiveness of counseling for smoking cessation and boosting their self-confidence in providing tobacco counseling.

## Introduction

Smoking tobacco is a prevalent global chronic disease resulting from nicotine dependence, posing a major public health challenge with nearly eight million deaths annually.^1^ It is the leading preventable cause of premature death and it is associated with increased risks of respiratory and cardiovascular diseases. It is also linked to over 20 types of cancer,^2,3^ among which, squamous cell carcinoma is the most prevalent oral cancer type.^4^

Brazil is the world's third-largest tobacco producer, and Rio Grande do Sul, the southernmost state in the country, is the leading producer of leaf tobacco.^5^ Approximately 9.3% of Brazilian adults smoke (11.7% of men and 7.2% of women), and cigarette experimentation typically begins at age 16.^6,7^

Smoking rates have declined due to tobacco control measures, including public health education, advertising restrictions, and smoke-free regulations.^7–9^ Brazil's National Tobacco Control Program has implemented smoking cessation interventions since 1996, and tobacco cessation treatment has been offered through the healthcare system since 2002. These interventions range from brief counseling to intensive cognitive-behavioral counseling and drug therapies such as nicotine replacement and bupropion.^6,7,10,11^

Dentists play a pivotal role in identifying smokers, managing tobacco-related oral lesions, and providing tobacco cessation counseling (TCC). Because of their interaction with patients from different age groups and the number and frequency of visits they receive, dentists benefit from a special dentist-patient relationship.^12^ Their involvement in tobacco treatment may positively impact smoking reduction.^13,14^ Nevertheless, many dentists and dental students report lacking sufficient knowledge, training, time, or resources to effectively implement such practices.^15–17^

Continuing education activities (CEAs) have been implemented to enhance the skills and knowledge of healthcare professionals, particularly in managing oral mucosal lesions and oral cancer.^18,19^ The Projeto Maio Vermelho ("Red May Project") is a notable collaborative effort between health departments and dental schools in Rio Grande do Sul. The project has conducted annual training on oral cancer prevention and detection since 2011. The aim of the present study was to evaluate the knowledge, attitudes, and perceptions of dentists and dental students who attended a CEA organized by the Projeto Maio Vermelho.

## Methods

### Study design, ethical considerations, and sampling

This was a pre-/post-intervention study consisting of a pre-intervention questionnaire, an educational intervention, and a post-intervention questionnaire. The present protocol was evaluated and approved by the local research and ethics committee (GPPG/HCPA no. 2021-0417), and the study complied with the Declaration of Helsinki. Participants were selected from a convenience sample of professionals and dental students attending the CEA "Smoking cessation: How does the dentist participate in this decision?" organized by the Projeto Maio Vermelho in May 2021. Registration for the CEA was conducted on the TelessaúdeRS-UFRGS's platform, a project in which specialists from different areas provide clinical support to primary healthcare professionals.^20^ The CEA was promoted on the Instagram page of the Projeto Maio Vermelho and the live session was streamed on TelessaúdeRS-UFRGS's YouTube channel (https://www.youtube.com/c/TelessaudeRS).

### Inclusion/exclusion criteria

Dentists and undergraduate dental students who attended the CEA and completed the questionnaires before and after the educational intervention were eligible for the study. Participants with missing data (age, sex, and/or professional category) were excluded from the study.

### Interventions

The educational intervention lasted 90 minutes and was conducted in an interview format. The topics included smoking as a disease, epidemiological data, effect of tobacco on the oral cavity, nicotine addiction, treatment methods, and the role dentists in smoking cessation. Participants were encouraged to actively participate by asking questions or making comments via the YouTube chat box.

Questionnaires were applied before and after the educational activity to assess knowledge, attitudes, and perceptions of the participants regarding TCC and treatment. The pre-intervention questionnaire consisted of 20 close-ended questions, whereas the post-intervention questionnaire contained nine close-ended questions.

### Statistical analysis

Descriptive statistics were used for describing the sample characteristics. Data distribution was assessed using the Shapiro-Wilk test. Experiences, behaviors, and perceptions of the participants were compared according to educational level, type of dental school, and years since graduation using the chi-square test. Years since graduation were dichotomized by the median (8 years). Analyses were performed using SPSS (version 25.0) and GraphPad Prism 8 software (Dotmatics, California, USA) and the significance level was set at 5%.

## Results

A total of 99 participants answered the pre-intervention questionnaire, but five were not included in the analysis because they were from other professional categories. Therefore, 94 participants were included. A total of 56 participants answered the post-intervention questionnaire, but four were excluded because of missing data, yielding a total of 52 participants (55.3%). The sample characteristics are shown in Table 1.

**Table 1:** Characteristics of the sample (n = 94).

Variable		Undergraduate students	Dentists	Total
Age (years)	
	Mean (SD)	24.1 (7.1)	35.2 (9.4)	31.2 (10.2)
	Min–Max	19-47	23-56	19-56
Sex				
	Male, n (%)	3 (8.6)	5 (8.5)	8 (8.5)
	Female, n (%)	32 (91.4)	54 (91.5)	86 (91.5)
Educational level				
	Undergraduate students, n (%)	35 (100)	-	35 (37.2)
	Non-graduate dentists, n (%)	-	19 (32.2)	19 (20.2)
	Graduate dentists, n (%)	-	40 (67.8)	40 (42.6)
Type of school				
	Public, n (%)	16 (45.7)	39 (66.1)	55 (58.5)
	Private, n (%)	19 (54.3)	20 (33.9)	39 (41.5)
Setting (n = 59)				
	Public, n (%)	-	39 (66.1)	39 (66.1)
	Private, n (%)	-	6 (10.2)	6 (10.2)
	Both, n (%)	-	14 (23.7)	14 (23.7)
Years since graduation (n = 59)				
	Mean (SD)	-	11.1 (9.5)	11.1 (9.5)
	Min–Max	-	1-34	1-34
	Median (P25-P75)	-	8 (3-18)	8 (3-18)

Table 2 shows that most participants usually ask patients about smoking habits (70.2% usually use the question "Do you smoke", with "Have you ever smoked" accounting for 26.6%). Only 3.2% reported not asking about smoking habits as part of their practice. Passive tobacco exposure, on the other hand, is not frequently addressed. All participants ask about cigarette use when assessing patients’ tobacco intake, whereas few participants ask about other forms of tobacco use, such as cigars, pipes, waterpipes, smokeless tobacco, or electronic cigarettes. Advice about the risks of tobacco intake is often provided, but few participants usually ask about the degree of dependence or readiness to stop smoking.

**Table 2:** Practices of undergraduate dental students and dental professionals regarding tobacco cessation counseling (TCC) (n = 94).

Practice items		n	%
How do you ask your patients about their smoking habits?			
	Do you smoke	66	70.2
	Have you ever smoked	25	26.6
	Do not usually ask about it	3	3.2
About what tobacco and nicotine products do you inquire directly?			
	Cigarette	94	100.0
	Cigars	20	21.3
	Pipes	21	22.3
	Smokeless tobacco	17	18.1
	Water pipes / hookahs	24	25.5
	Bidis	3	3.2
	Electronic cigarettes	30	31.9
Do you explain to patients the risks associated with tobacco?			
	Always	38	40.4
	Almost always	28	29.8
	Sometimes	25	26.6
	Rarely	1	1.1
	Never	2	2.1
Among the items below, which ones do you include in your anamnesis about smoking habits?			
	Frequency of cigarettes/day	89	94.7
	Quitting smoking attempts	48	51.1
	Utilized products	42	44.7
	Readiness to quit smoking	26	27.7
	Degree of dependence	20	21.3
Do you usually ask your patient about passive smoking?			
	Always	7	7.4
	Almost Always	13	13.8
	Sometimes	13	13.8
	Rarely	27	28.7
	Never	34	36.2

TCC is not commonly addressed in dental school, considering that most participants reported not having such training during their undergraduate studies (67%) or after graduating (71.2%). Moreover 70.2% of participants consider that TCC is not effective because of the lack of formal education on the subject (Table 3). Despite the lack of training and knowledge about assessing the degree of dependence on nicotine, most participants mentioned providing patients with some level of counseling on smoking cessation. Furthermore, 96.2% of participants showed interest in receiving formal training on tobacco treatment and were motivated to counsel patients to stop smoking (Table 3). In relation to tobacco cessation strategies, most participants (n=83, 88.3%) stated that combining cognitive-behavioral and pharmacological approaches is more efficient.

**Table 3:** Training, attitudes, and perceived effect items of undergraduate dental students and dental professionals towards tobacco cessation counseling (TCC) (n = 94)

Variables			n	%
TCC training items				
	Have you attended TCC training during your undergraduate studies?			
		Yes	31	33.0
		No	63	67.0
	Have you attended TCC training after graduation?			
		Yes	17	28.8
		No	42	71.2
	Would you be interested in having formal TCC training?			
		Yes	50	96.2
		No	2	3.8
Attitude items			
	Do you usually counsel patients to quit smoking?			
		Always	21	22.3
		Almost Always	25	26.6
	Sometimes		33	35.1
		Rarely	8	8.5
		Never	7	7.4
	Do you know how to ask about the degree of nicotine dependence?			
		Yes	23	24.5
		No	71	75.5
	How long do you believe would be a reasonable time to perform TCC?			
		Less than 5 minutes	28	29.8
		Approximately 15 minutes	41	43.6
		At least 30 minutes	23	24.5
		Around 60 minutes	2	2.1
	How do you imagine smoker patients would react on receiving information about tobacco hazards or TCC?			
		Dissatisfied	14	14.9
		Indifferent	38	40.4
		Satisfied	42	44.7
	After this CEA, do you feel motivated to perform TCC?			
		Yes	50	96.2
		No	2	3.8
Perceived effect items			
	Which approach do you believe is more efficient for tobacco cessation?			
		Cognitive-behavioral approach	11	11.7
		Pharmacological approach	0	0
		A combination of both	83	88.3
	Public universities			
		Cognitive-behavioral approach	2	5.6
		Pharmacological approach	0	0
		A combination of both	53	96.4*
	Private universities			
		Cognitive-behavioral approach	9	23.1
		Pharmacological approach	0	0
		A combination of both	30	76.9*
	Do you think TCC is not effective due to lack of formal training?			
		Yes	66	70.2
		No	28	29.8

Chi-square test, asterisks indicate statistically significant associations (p < 0.01).

### Type of school

Participants from public institutions were more likely to perceive the combination of cognitive-behavioral therapy with drug treatment as the most effective smoking cessation strategy. Among undergraduate dental students or dentists from public universities, 96.4% considered the use of both approaches as the most efficient treatment, compared to 76.9% among those from private institutions (p < 0.01) (Table 3).

### Degree of education

Currently undergraduate dental students have easier access to TCC training (48.6%) than non-graduate (21.1%) or graduate dentists (25%) (p = 0.04). Graduate dentists had higher frequency of TCC training (37.5%) than non-graduate dentists (10.5%) (p = 0.03) (Table 4).

**Table 4:** Comparison of access to training, practices, attitudes, and perceptions in relation to tobacco cessation counseling (TCC) according to participants’ qualification (% of answers).

Variables				Undergraduate dental students	Non-graduate dentists	Graduate dentists	p-value
				(n = 35)	(n = 19)	(n = 40)	
Questions							
	Access to TCC training items						
		During undergraduate studies					
			No	51.4	78.9	75.0	0.04
			Yes	48.6*	21.1	25.0	
		During professional life					
			No	–––	89.5	62.5	0.03
			Yes	–––	10.5	37.5*	
Practice items							
	Previous attempts to quit smoking						
		No		34.3	78.9*	47.5	< 0.01
		Yes		65.7*	21.1	52.5	
	Products used						
		No		42.9	84.2*	52.5	0.01
		Yes		57.1	15.8	47.5	
Perception item							
	Time necessary to conduct TCC						
		At least 5 min		40.0	52.6*	10.0	< 0.01
		About 15 min		45.7	31.6	47.5	
		At least 30 min		11.4	15.8	40.0*	
		About 60 min		2.9	0.0	2.5	

Chi-square test, asterisks indicate statistically significant associations

Non-graduate dentists seldom ask patients about the different forms of tobacco consumption. Also, most of these participants (52.6%) consider 5 minutes adequate to perform TCC, whereas graduate dentists generally believe that at least 30 minutes is needed. An association was also observed between undergraduate students and a higher frequency of asking about previous attempts to stop smoking (Table 4).

### Years since graduation

Dentists with 8 or more years since graduation reported having attended TCC training more frequently (41.3%) than those who graduated more recently (13.8%) (*p* < 0.01). Also, more experienced dentists reported asking patients more often about the frequency of cigarettes smoked per day, different forms of tobacco intake, attempts to stop smoking, and passive smoking exposure. They also felt more prepared to assess the degree of nicotine dependence (Table 5).

**Table 5:** Comparison of access to training, practices, and attitudes in relation to tobacco cessation counseling (TCC) according to years since graduation (% of answers).

Questions			< 8 years	≥ 8 years	p-value
			(n = 27)	(n = 32)	
Access to TCC training items					
	During undergraduate studies				
		No	86.2*	58.7	< 0.01
		Yes	13.8	41.3*	
Practice items					
	Frequency of cigarettes/day				
		No	13.8*	1.6	0.02
		Yes	86.2	98.4*	
	Attempts to quit smoking				
		No	69.0*	38.1	< 0.01
		Yes	31.0	61.9*	
	Products used				
		No	79.3*	46.0	< 0.01
		Yes	20.7	54.0*	
	Capacity to ask about level of dependence				
		No	93.1*	71.4	0.02
		Yes	6.9	28.6*	
	Passive smoking exposure				
		Never	55.2*	28.6	0.03
		Hardly ever	27.6	27.0	
		Sometimes	13.8	14.3	
		Often/Ever	3.4	30.1*	

Chi-square test, asterisks indicate statistically significant associations.

### Impact of CEA

Figure shows the impact of the CEA on participants’ perception regarding TCC. The general opinion of participants on TCC was very positive, even before the CEA. Most felt that the dental clinical setting is appropriate for tobacco counseling and considered that effective TCC and motivational counseling by a dentist can help patients stop smoking.

**Figure:**
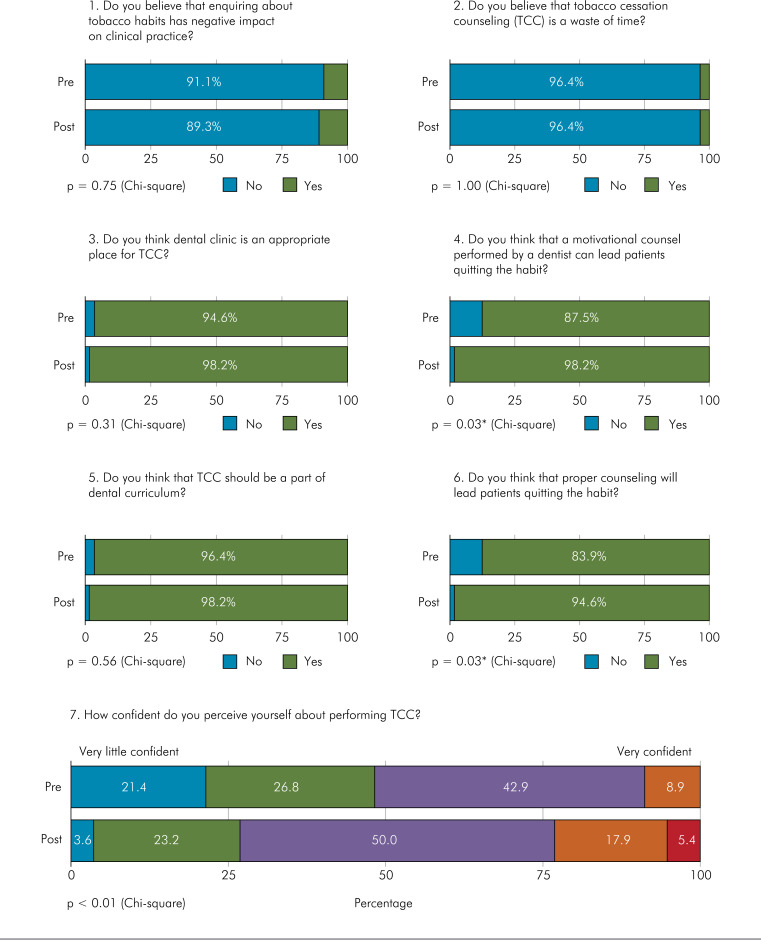
Perceptions of dental professionals and students regarding tobacco cessation counseling (TCC).

After a brief CEA on the subject, participants showed greater confidence in the possibility of counseling effectively result in smoking cessation (pre-intervention, 87.5%; post-intervention, 98.2%; p = 0.03). In addition, the evaluation showed an increase in the number of participants who felt confident in performing TCC (from 8.9% to 23.3%; p < 0.01).

## Discussion

This study aimed to evaluate the knowledge, attitudes, and perceptions of CEA attendees regarding smoking. The findings indicate that participation in CEA enhanced the awareness of dentists and dental students about smoking habits and risks. Also, these professionals acknowledged their vital role in assisting patients with smoking cessation. Formal TCC training is not commonly integrated into dental undergraduate and graduate programs, a gap that participants believe could undermine the effectiveness of TCC. However, participants expressed a keen interest in receiving formal training on TCC. The brief CEA yielded promising outcomes, reinforcing dentists’ and dental students’ belief in the effectiveness of counseling for smoking cessation while boosting their self-confidence in providing TCC.

The Guideline for Treating Tobacco Use and Dependence suggests that the initial step in addressing this issue is to identify tobacco users.^21^ The guideline recommends assessing every patient for tobacco use and their willingness to quit smoking. The first three steps of the five A's model (ask, advise, and assess) are very helpful in this process. Most participants (96.8%) reported routinely inquiring about smoking habits, as shown by previous studies.^22–24^ Many of the participants, however, usually question about current smoking habits ("Do you smoke?") and not about past smoking habits ("Have you ever smoked?"), potentially overlooking patients who have quit smoking. Moreover, dentists were not as vigilant about alternative methods of tobacco exposure, such as electronic cigarettes and waterpipes, which have gained popularity, particularly among the younger population.^25,26^

While e-cigarettes are marketed as a cessation aid,^27^ their efficacy remains uncertain.^28^ In young individuals, they may even serve as a gateway to other forms of smoking.^29^ The long-term effects of e-cigarettes are still unknown.^26,28^ Recent studies have indicated signs of cytotoxicity and genotoxicity in e-cigarette users.^30,31^ Therefore, it is important to inquire about this habit routinely. The same applies to passive smoking, another critical aspect that is often overlooked, contributing to approximately 1.2 million deaths annually.^8^

Participants regarded the dental clinical setting as suitable for providing TCC, which is consistent with the findings of other studies.^23,32^ Most participants believed that TCC would not negatively impact their clinical practice, with nearly 45% stating that patients would welcome tobacco-related information or TCC. These findings reinforce those described in previous studies,^33,34^ which reported an association between smoking cessation interventions and increased patient satisfaction with their care and positive perception of TCC delivery in the dental clinical setting. Conversely, studies by Al-Maweri et al.^23^ and Koka et al.^22^ indicated that many dentists and dental students perceived smoking cessation interventions as potentially detrimental to their practice and income. Discussions on smoking hazards are frequently incorporated into undergraduate programs, as reported by Leonel et al.^35^ This could explain why most participants in this study discussed the risks of smoking with patients and attempted to persuade them to quit.

A study by Strey et al.^36^ found that 87.5% of participants frequently provided TCC, in which is comparable to the findings of our study (84.0%). However, Chaffee et al.^13^ and Koka et al.^22^ reported that dentists routinely inquire about smoking habits but fail to intervene. Insufficient training and knowledge were identified as possible reasons for that.^15,17,23,24,32^ Recently, an increasing number of studies have highlighted the valuable role of dental professionals in supporting smoking cessation efforts.^14,37^ Our findings indicate that only a small proportion of participants received formal training on TCC at dental school or after graduation. Similar outcomes were reported by Leonel et al.^35^ Nonetheless, 96.2% of participants in our study were keen on receiving formal TCC, in accordance with the literature.^15,17^ The inclusion of smoking cessation training in the dental curriculum receives strong support from the participants of this and other studies.^23,24,32^

CEAs have proven effective in raising awareness of oral cancer.^18^ Previous studies from our research group also revealed interesting results, including increased self-efficacy for managing oral cancer after a theoretical section on oral diagnosis.^19^ In this study, the brief CEA on TCC enhanced the perception of dentists and dental students regarding the effectiveness of TCC. This finding aligns with Kachwaha,^24^ who reported that most participants believe that TCC provided by dentists can help patients quit smoking.

In line with earlier studies, CEAs on smoking cessation positively influenced self-confidence in providing TCC.^38,39^ To fill the current gap, undergraduate programs should prioritize incorporating basic training on smoking cessation in the core curriculum, considering that most schools presently lack such courses. This also applies to establishing regular training programs for health professionals in both public and private care settings.

Among professionals, more experienced dentists reported greater participation in smoking cessation training and a higher frequency of patient inquiries about smoking and nicotine dependence compared to less experienced dentists. Alajmi et al.^40^ identified a correlation between more experienced professionals and increased willingness to conduct tobacco cessation activities, which is likely attributed to professional maturity, financial stability, and career development. Conversely, Al-Maweri et al.^23^ found that recent graduates displayed better practices and attitudes toward TCC compared to more experienced professionals, possibly due to declining knowledge and motivation over time, thus underscoring the importance of frequent CEAs.

This study has some limitations. Our sample included dentists and dental students who voluntarily attended a CEA, making it challenging to extrapolate the results to a larger population. Studies indicate that health professionals who smoke may be less inclined to engage in TCC training.^15,23,40^ Another limitation is that smoking habits were not assessed in this study. Additionally, participants who attend CEAs, such as the one proposed by the Projeto Maio Vermelho, may be more proactive in seeking knowledge, potentially leading to an overestimation of the beliefs and attitudes regarding TCC presented in this study.

## Conclusion

Undergraduate dental students and dentists showed awareness of patients’ smoking status and engagement in TCC, despite the lack of formal training. Our findings suggest that a brief CEA on TCC may improve the perception of dentists and undergraduate dental students of the effectiveness of counseling on smoking cessation and boost their self-confidence in providing TCC. It is essential to include basic TCC training in dental curricula and in CEAs for dental professionals, given that tobacco use is extremely harmful to the oral and general health.
